# Assessing social–ecological feedbacks in small-scale fisheries

**DOI:** 10.1007/s13280-026-02407-w

**Published:** 2026-05-14

**Authors:** Leandro Castello, Adriana Guimarães Abreu, George Christopher Brooks, João Vitor Campos-Silva, Priscila dos Reis Cunha, Ayan Santos Fleischmann, Caetano Lucas Borges Franco, Ana Claudia Gonçalves, Emma Ann Hultin, Holly Kathleen Kindsvater, Eduardo Guimarães Martins, Cristina Isis Buck Silva, Michael G. Sorice, Eduardo Sonnewend Brondizio

**Affiliations:** 1 Department of Fish and Wildlife Conservation, Virginia Tech, Blacksburg, VA USA; 2 Department of Anthropology, Indiana University, Bloomington, IN USA; 3 INPA, Programa de Pós-Graduação em Ecologia, Instituto Nacional de Pesquisas da Amazônia, Instituto Juruá, Manaus, Amazonas Brazil; 4 Department of Forest Resources and Environmental Conservation, Virginia Tech, Blacksburg, USA; 5 Department of Ecosystem Science and Management, University of Northern British Columbia, Prince George, BC Canada; 6 Instituto de Filosofia e Ciências Humanas, Campinas, Brazil; 7 Present Address: Center for Limnology, University of Wisconsin-Madison, Madison, USA; 8 Mamirauá Sustainable Development Institute, Tefé, Amazonas Brazil

**Keywords:** Co-management, Fisheries assessment, Small-scale fisheries, Social–ecological systems, Sustainability

## Abstract

Assessment frameworks do not capture the complexity of the social–ecological dynamics of small-scale fisheries (SSF), which support millions of livelihoods yet face persistent sustainability challenges. Reciprocal feedbacks between fish populations and fishers that are central to sustainability remain insufficiently integrated into assessment approaches because conventional fisheries management emphasizes population dynamics, whereas social–ecological systems research focuses on social drivers and faces operational challenges. We propose an integrative diagnostic approach that explicitly links fish population dynamics with theories of fisher behavior and governance. We illustrate its application using co-managed arapaima fisheries in the Amazon Basin, where sustainability emerges from multi-scalar social and ecological interactions. By capturing these feedbacks, the approach bridges ecological and social dimensions to identify key drivers of sustainability. It provides a replicable, interdisciplinary framework for diagnosing SSF sustainability and identifying leverage points to support adaptive governance across diverse contexts.

## Introduction

Fishing involves complex social–ecological interactions that challenge the sustainability of fisheries (FAO 2024a). Fishing alters fish populations, and subsequent changes in fish abundance, market value, or management conditions feedback to influence fishers’ cooperation and harvesting behavior. Difficulties in managing these feedbacks have contributed to one-third of industrial fisheries being fished beyond biologically sustainable levels (FAO 2024b). These challenges are even more acute in artisanal, small-scale fisheries (SSF, sensu Smith and Basurto 2019), which comprise most fisheries systems and produce 40% of the global fish catch (FAO 2022). Compared with large-scale industrial fisheries, SSF tend to face higher risks of overfishing because management capacity, enforcement, and data availability are often more limited (Andrew et al. 2007; Costello et al. 2012; Worm and Branch 2012). However, existing assessment frameworks do not adequately capture the reciprocal social–ecological feedbacks that underpin the sustainability of SSF.

SSF are vulnerable to endogenous and exogenous factors and processes (Salas et al. 2007; Basurto et al. 2025) that operate at different temporal and spatial scales (Bohensky and Lynam 2005). Most SSF are in tropical developing nations where governments typically lack management resources and fishers often belong to the poorest and most marginalized groups of society (Pauly 1997; Funge-Smith and Bennett 2019). In these contexts, SSF are affected endogenously by fishers’ dependence on the resource, competition among fishers with unequal access to technology, and weak institutional capacity of public agencies formally responsible for management and enforcement (Dugan et al. 2006; Islam and Chuenpagdee 2022). These factors can reinforce vulnerability by increasing pressure on fish stocks while weakening collective management capacity. Further vulnerabilities to SSF come from external political, institutional, and economic forces that can override local-level management, for example through subsidies or legislation that distort incentives and increase economic pressure on fishers (Cochrane and Doulman 2005). Somewhat unique to SSF are biophysical influences that manifest at small spatial scales (e.g., coral habitat structure) that influence fish populations through heterogeneous mosaics of environmental conditions (Welcomme et al. 2010; Castello et al. 2018).

Accounting for these vulnerabilities in the assessment of SSF requires approaches that consider both the social and ecological dimensions at multiple scales (Smith et al. 2021). The conventional approach to fisheries management has largely relied on modeling of the dynamics of fish populations to inform management rules, and implementation of management rules to control fishers’ behavior (Hilborn 2007; Fulton et al. 2011). Because this approach has struggled to foster sustainability, the paradigm has been shifting toward understanding fisheries as integrated social–ecological systems (Mahon 1997; Berkes et al. 2001; Andrew et al. 2007). Within this paradigm shift, two schools of thought have made substantial contributions. The social–ecological systems (SES) approach pioneered by Ostrom (2007, 2009) provides a diagnostic framework for examining how institutional arrangements, property rights, and collective action shape resource use. The coupled human and natural systems (CHANS) approach pioneered by Liu et al. (2007, 2021) draws on complexity science to highlight nonlinear feedbacks, teleconnections, and emergent properties in linked human–environment systems. Both approaches address limitations of the conventional fisheries paradigm by analyzing SSF together with the endogenous and exogenous processes influencing ecological and socioeconomic dynamics (McClanahan et al. 2009; Kittinger et al. 2013; Liu et al. 2021).

However, disciplinary divides and the absence of meaningful interdisciplinary dialogue (Pauly 2006; Vogt et al. 2015) have limited consideration of key social and ecological interactions in fisheries. The focus of the conventional approach on the effects of fishing on fish populations has led it to inadequately account for the social, cultural, and institutional processes that shape fishers’ behavior (Jentoft 2006). While both the SES and CHANS approaches conceptually emphasize integrated social–ecological dynamics, their implementation has been uneven. SES studies have often focused on the social conditions that enable sustainable fishing while insufficiently accounting for ecological dimensions (Epstein et al. 2013; Vogt et al. 2015). Similarly, CHANS studies have emphasized complex system properties but faced challenges in operationalizing behavioral responses and validating coupled dynamics empirically (Nielsen et al. 2018; Jones et al. 2019).

Consequently, understanding of the reciprocal feedbacks between fish populations and fisher behavior that shape system dynamics remains limited. Very few studies have explicitly accounted for these two-way interactions that allow fisheries to self-regulate and remain sustainable (Edmondson and Fanning 2022; Charles 2023). In particular, while fishing effects on fish populations are relatively well understood, much less is known about how fishers respond to changes in fish populations (Salas and Gaertner 2004; Hilborn 2007; Fulton et al. 2011). Addressing this gap requires approaches that explicitly account for reciprocal fish–fisher interactions as well as broader social and ecological drivers (Schlüter et al. 2019), and that can be operationalized in real-world SSF contexts.

To fill this gap, we present an integrative diagnostic approach that links fish population dynamics with theories of fisher behavior and governance, and we conceptually illustrate its application to a SSF. Our approach explicitly incorporates the reciprocal interactions between fish and fishers as well as multi-scalar social and ecological drivers. By synthesizing key insights from conventional fisheries science and SES research, our approach provides an interdisciplinary and conceptually rigorous framework for diagnosing SSF sustainability. By integrating ecological and social science with practitioner knowledge, the approach also aligns with broader transdisciplinary efforts in sustainability research. Importantly, the approach emphasizes identifying key feedback mechanisms and leverage points rather than attempting exhaustive characterization of system variables. The term *‘assessment’* here is analogous to *‘diagnosis’* and refers to defining key sustainability challenges within a nested, coupled system. In line with the systemic sustainability perspective (Andrew et al. 2007; Ostrom 2009; Folke et al. 2016), it involves identifying the ecological dynamics that underpin social and economic vulnerabilities, thereby revealing the key levers of sustainability and informing future governance and economic responses. By *‘levers,’* we mean the specific ecological, social, or institutional factors that, when changed, can shift system dynamics toward or away from sustainability (Fischer and Riechers 2019). Identifying such levers is essential for designing targeted governance strategies in the absence of extensive data or enforcement capacity.

We describe how a group of collaborating researchers and managers with expertise in the fields of fish ecology, fisheries, hydrology, anthropology, social psychology, fish population dynamics, and policy is attempting to integrate social–ecological feedbacks in SSF for arapaima (*Arapaima gigas*, *A. agasizzii*, *A. mapae*, *A. leptosoma*, and *A. arapaima*; Stewart 2013a, b). SSF for arapaima are emblematic cases of co-management of highly overexploited fisheries in the Amazon Basin. In line with the SES approach of Ostrom (2009) and the CHANS approach (Liu et al. 2021), our approach views SSF for arapaima as nested, multi-scalar social–ecological systems, comprising fish populations, floodplain lakes, river hydrology, community and government institutions, fishers’ cultural perspectives, and regional markets, policies, and governance. The approach builds on the conventional fisheries and SES approaches and incorporates the novel framework proposed by Schlüter et al. (2019) that puts social–ecological interactions at the center of analysis to explain the emergence of sustainability.

Below, we assess the adequacy of fisheries and SES approaches for SSF (Approaches to understand small-scale fisheries) and show that Schlüter et al.’s framework offers a starting point to address their limitations. We then describe the case of arapaima SSF, including the evolution of co-management and current dilemmas (The case of arapaima small-scale fisheries). We next introduce our main contribution—an integrative approach to assess reciprocal fish–fisher feedbacks—and conceptually illustrate its application to arapaima SSF (Integrating social–ecological feedbacks), including outlining pathways for future real-world operationalization (Toward operationalizing the approach). Throughout the paper, we deliberately minimize the use of disciplinary jargon to foster interdisciplinary integration. Just as we refer to ecological dimensions to capture the biotic and abiotic aspects of the natural environment, we use the term social dimensions to inclusively refer to institutional, cultural, and economic aspects. By weaving together critical social–ecological interactions, our approach helps uncover the levers of sustainability to inform policy and management.

## Approaches to understand small-scale fisheries

**Fig. 1:**
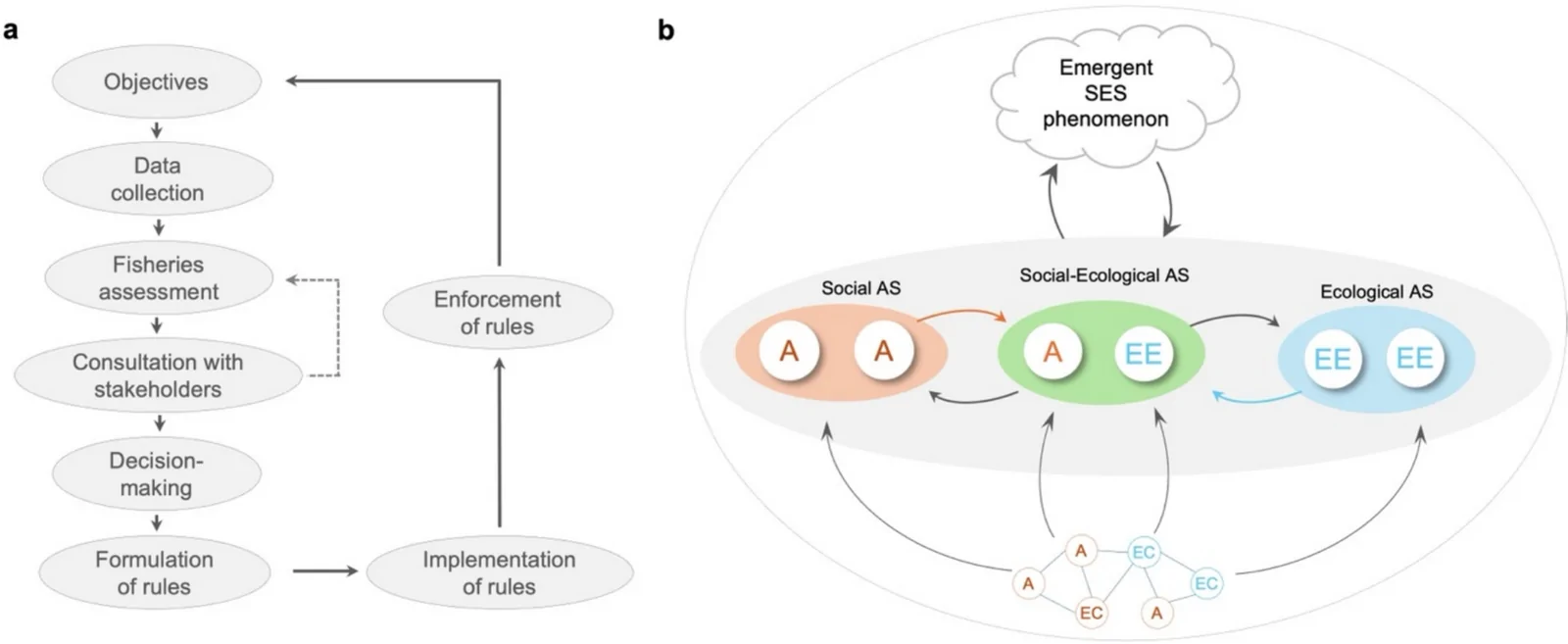
**a** Diagrammatic representation of the main functions and responsibilities involved in the conventional approach to fisheries assessment and management, as proposed by the Fisheries Department of the Food and Agriculture Organization of the United Nations (adapted from Cochrane (2002). **b** Schematics of the social–ecological action situation (SE-AS) framework (adapted from Schlüter et al. 2019) designed to capture the reciprocal social and ecological interactions that are responsible for the emergence of SES phenomena. The gray ellipse in the middle represents a configuration of AS that includes the social action situation (S-AS), ecological action situation (E-AS), and the social–ecological action situation (SE-AS), where the E-AS and S-AS interact. This configuration of action situations (AS) is developed by identifying those social and ecological interactions in the SES that are thought to be critical drivers of change, and that are under the influence of other social and ecological factors, processes, and interactions that can include actors (A) and ecosystem components (EC)

The conventional fisheries and SES approaches have distinct and opposing strengths. The conventional fisheries approach views sustainability primarily as an ecological issue. It focuses on regulating fishing practices to ensure fish catch does not exceed the biological productive capacity of exploited fishery stocks (Cope 2024). In this approach, management agencies collect and analyze biological and ecological data to assess fishery stock exploitation status. With the aim of maximizing socioeconomic benefits (e.g., maximum sustainable yields), scientists propose sustainable targets, which government bodies translate into fishing regulations and enforce on fishers (Cope 2024; Fig. 1a).

The conventional fisheries approach performs well in contexts with sufficient human and financial resources, such as developed nations, and in fisheries with high economic value, which are typically large-scale and industrial (Hilborn et al. 2020; Melnychuk et al. 2021). Its success in these cases stems from the ability to monitor and control fishing effort, assess stock status, and enforce regulations. These management functions are supported by the theoretical foundation of population ecology, which describes how fish populations respond to environmental variability and exploitation (Jennings et al. 2009). Empirical studies have consistently shown that fishing intensity, timing, and size selectivity affect the capacity of fish populations to be sustainably harvested (Quinn and Deriso 1999). Accordingly, management rules commonly aim to regulate fishers’ behavior by setting quotas, size limits, and seasonal closures (Dowling et al. 2015).

The conventional fisheries approach is less effective in contexts with weak governmental capacity and low-value fisheries, where the costs of implementation outweigh perceived benefits (Pauly 2006; Salas et al. 2007; Ruddle and Hickey 2008). It also tends to overlook local knowledge and the social dynamics that influence fishing practices and compliance (Berkes et al. 2000; Turner et al. 2008). As a result, the approach has been only partially implemented in most SSF, which are in developing nations, with governments often adopting limited rules while rarely assessing stocks or enforcing regulations (Mahon 1997). Even when fully implemented, it has largely failed to address the underlying drivers of overfishing that lie outside its institutional and conceptual scope, such as the social, economic, and perceptual factors that shape fisher behavior, which typically lie outside its institutional and conceptual scope (Andrew et al. 2007; Cánovas-Molina and García-Frapolli 2022).

A key reason for the growth of SES approaches is their ability to explicitly account for exogenous factors and processes affecting SSF that are not adequately accounted for in the conventional approach (Allison and Ellis 2001; Andrew et al. 2007). Diagnostic approaches based on SES, including livelihood approaches (e.g., Allison and Ellis 2001) and the SES framework of Ostrom (2007, 2009), now underpin state-of-the-art approaches for SSF research and governance. For example, the SES framework of Ostrom (2009) is used to frame SSF in the first module of the handbook of SSF governance published by the Food and Agriculture Organization of the United Nations (FAO 2024b). Ostrom’s SES framework organizes variables that characterize the resource system (e.g., fishery), resource units (e.g., fish), actors (e.g., fishers), and governance systems (e.g., rules, organizations). The SES framework was built upon Ostrom’s Institutional Analysis and Development Framework (IAD), which identifies sets of critical interacting variables that explain how fishers can self-organize to manage common pool resources (e.g., Cox et al. 2010a; Gutierrez et al. 2011; Cinner et al. 2012).

Despite its governance insights, SES research has not adequately accounted for ecological dynamics (Rissman and Gillon 2016; Smith et al. 2021). Although the SES framework includes ecological variables such as resource mobility and replacement rate, its architecture considers ecological dimensions scantly (Epstein et al. 2013; Vogt et al. 2015). The framework establishes that social–ecological interactions occur in what is termed the action situation (AS), which stems from a game theoretic model of institutional decisions (Ostrom 2011). This AS model assumes sustainability results mainly from social processes, considering ecological processes only when they pose decision-making challenges (Vogt et al. 2015).

To address these ecological gaps in SES research, recent advances have sought to integrate social and ecological dimensions more effectively, including the addition of new sub-systems for ecological rules and new ecological attributes (Epstein et al. 2013; Vogt et al. 2015). An important advance to capture key interactions that explain the emergence of system properties (e.g., fisheries sustainability) has been replacing the AS of the SES framework with the social–ecological action situation **(**SE-AS) proposed by Schlüter et al. (2019; Fig. 1b). In Schlüter et al.’s framework, the SE-AS is accompanied by a social action situation (S-AS) to capture purely social interactions (e.g., buying and selling), as well as an ecological action situation (E-AS) to capture relations or interactions between ecological or biophysical elements (e.g., fish habitat). Outcomes of one AS may affect other AS, so several ASs can be linked through a configuration of ASs, to account for varied and complex interactions demanding decision-making at different scales. By putting social–ecological feedbacks, not social dynamics, at the core of an analysis, Schlüter et al.’s framework goes beyond previous frameworks by focusing on the interplay of social–ecological interactions that explain sustainability (i.e., feedbacks among individual fishers, other community members, and ecological components within a fishery). As we show below for arapaima SSF, Schlüter et al.’s framework permits integrating fish population models from the conventional approach with variables characterizing fisheries from the SES framework to account for the feedbacks that explain sustainability.

## The case of arapaima small-scale fisheries

### The evolution of co-management

**Fig. 2:**
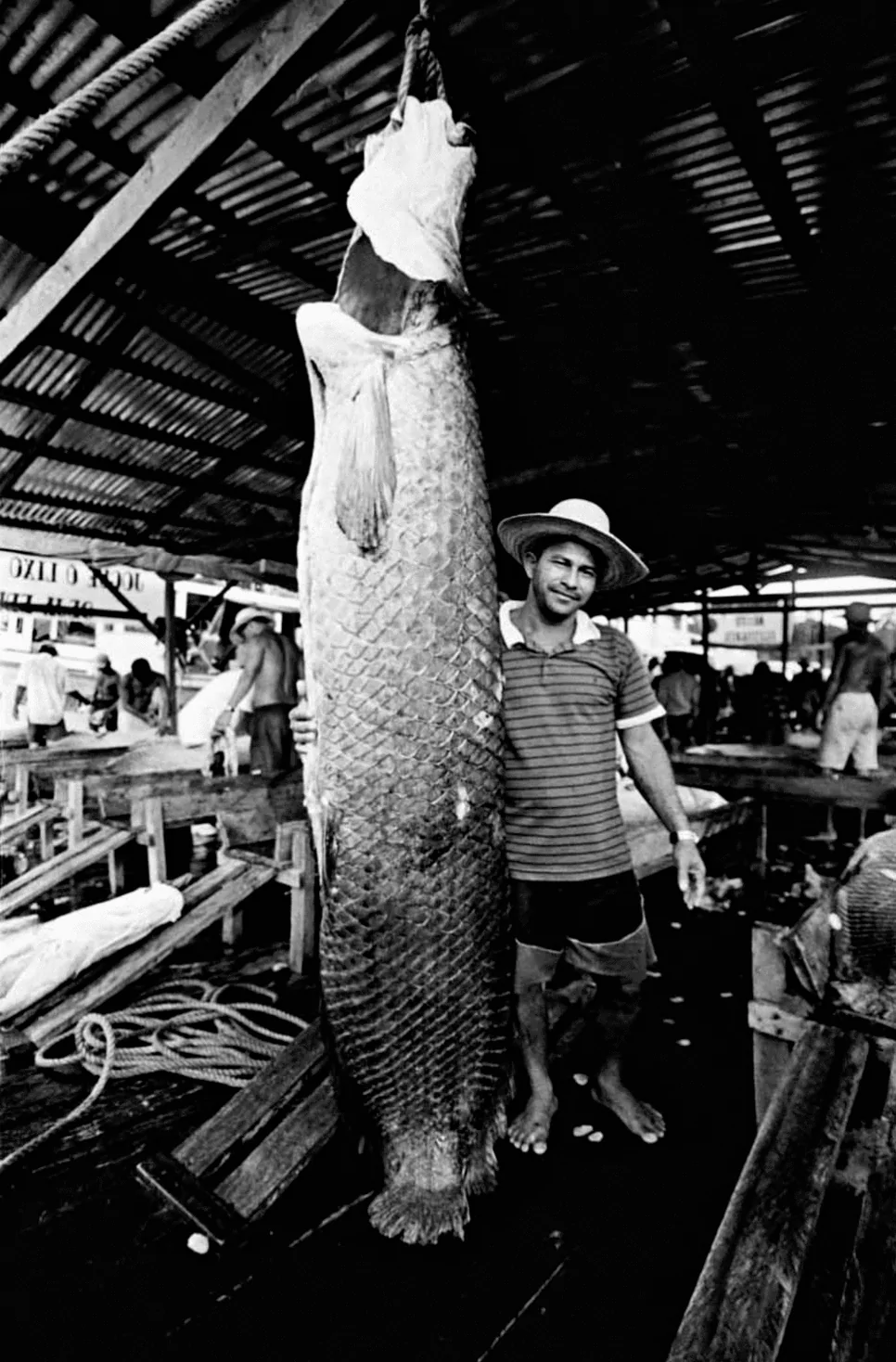
An arapaima fisher, João de Oliveira, who is involved in community-based management (CBM) agreement, seems proud of the catch. Large arapaima were rare 20 years ago, but are increasingly common, because of co-management. Photo credit: Castanheira (2011)

SSF for arapaima involve giant, freshwater fishes that can grow to three meters in length and 200 kg in weight (Fig. 2; Arantes et al. 2010; Watson et al. 2021). Arapaima are obligate air-breathers that surface every 5–15 min to gulp air (Stokes et al. 2021). This behavior makes them vulnerable to specialized fishers, who harpoon and/or set gillnets around them (Castello 2004).

Fishing for arapaima is a long-standing tradition in the Amazon Basin, dating back to pre-Columbian times (Prestes-Carneiro et al. 2016). Arapaima have historically fetched high market prices, and the skills and cultural meaning involved in harpooning highlight the identity of being an arapaima fisher (Veríssimo 1895; Lima et al. 1999; Gillingham 2001). Arapaima fishers are typically from riverine communities (locally known as *‘ribeirinhos’)* but also involve Indigenous groups and urban fishers (Alencar 2021).

Arapaima dominated Amazonian fisheries in the 1900s (Veríssimo 1895), but poor management made them widely overfished and even depleted in some regions (Castello et al. 2015a, b). In Brazil, adoption of the conventional approach to fisheries management led to implementation of rules of closed season (December–May) and minimum size of catch (1.5 m) for arapaima in the 1980 and 1990s, respectively (Viana et al. 2004). However, limited resources prevented governmental agencies from effectively enforcing these rules. As a result, illegal fishing became widespread, with up to two-thirds of the catch being illegal in some regions (Cavole et al. 2015). Limited resources hindered data collection and the application of most fisheries assessment methods (Castello 2004). As a result, information on exploited populations remained scarce, compounding the management challenge. This led the state management agency (Instituto Brasileiro do Meio Ambiente e dos Recursos Naturais Renováveis; IBAMA) to issue Ordinance No. 8 of February 2, 1996, effectively banning the fishery in the State of Amazonas, Brazil.

The decline of arapaima and other fisheries prompted ribeirinhos in the 1970s to establish partnerships with nongovernmental organizations to protect floodplain lake fisheries by developing community-based arrangements (Lima et al. 1999; McGrath et al. 2008). These arrangements complemented legislation by setting local-level fishing rules (e.g., gears allowed) and spatial zoning rules (e.g., classification of lake use) (Lima et al. 1999; Crampton et al. 2004a) that benefit fish populations and increase fish catch (Silva et al. 2025). The effectiveness of these arrangements prompted their incorporation within conservation units, which legally establish territorial spaces designed for nature preservation, and thus address a major dilemma by establishing common property rights for local fishers (Crampton et al. 2004a, b).

The importance of arapaima to ribeirinhos prompted a (then) nongovernmental organization (the Mamirauá Institute for Sustainable Development) to develop a co-management model in 1999. The model relied on the ability of expert fishers to count (i.e., census) surfacing arapaima (Castello 2004). In the model, every year fishers count the arapaima in their floodplain lakes, and, in conjunction with the Mamirauá Institute and IBAMA, use the data to set conservative harvest quotas for the following year (Viana et al. 2004). The fishers are granted a special authorization to harvest the quota by IBAMA under several conditions, including that they commit to abide by rules of size and season, develop community-level management rules, and patrol the lakes to enforce these rules.

**Fig. 3:**
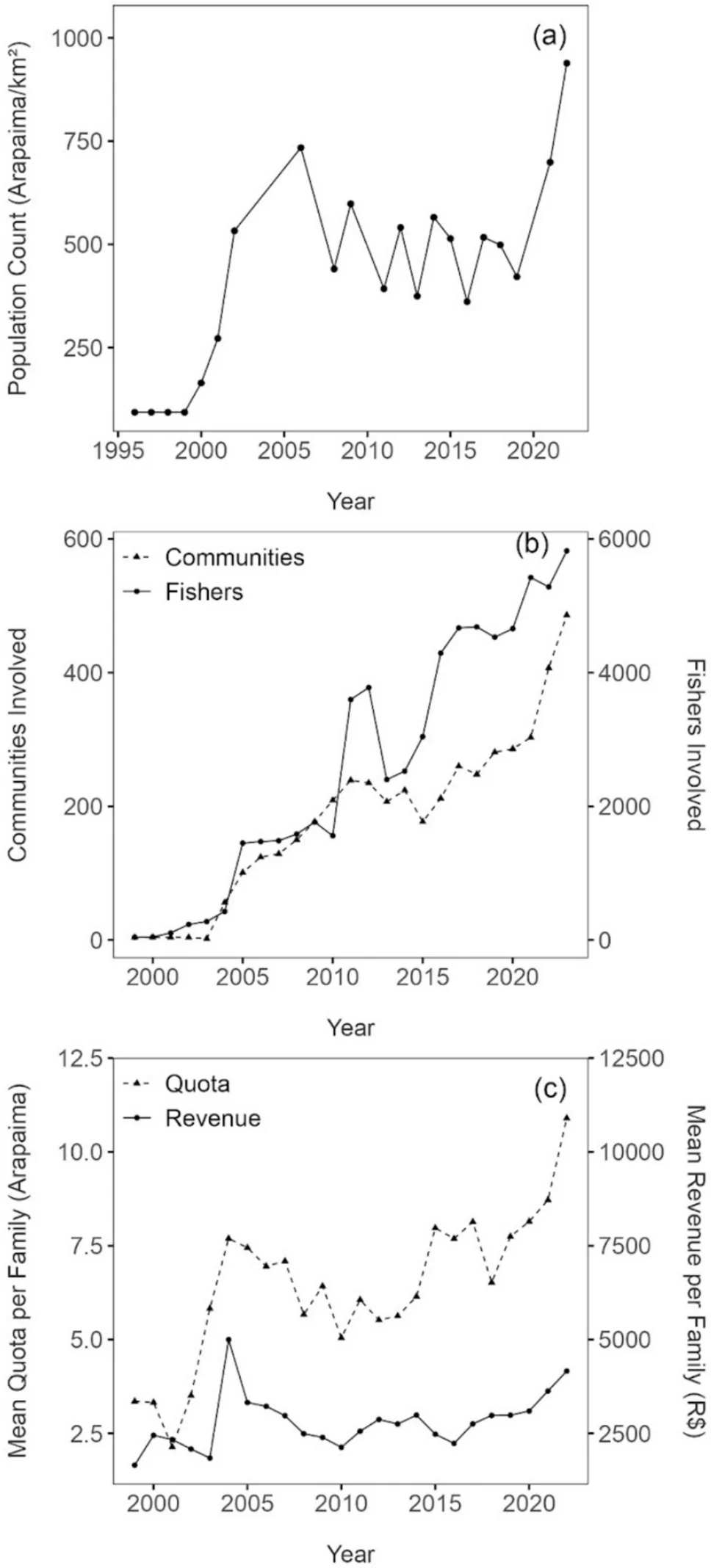
**a** Rapid recovery of the arapaima population in the first set of four fishing communities where the co-management model based on counts were first tested and implemented in 1999; the arapaima population for 1996–1999 is assumed to be the same as in 1999, when it was counted (see Castello et al. 2011). **b** Historical increase in numbers of fishing communities and fishers formally registered with the state management agency (IBAMA) to co-manage arapaima based on the counts. **(c)** Quota and revenue from arapaima co-management over time. The quota is measured in numbers of arapaima specimens fished and revenue is in inflation-corrected Brazilian Reais. Data are from annual reports submitted by fishing communities to IBAMA

The co-management model saw early success in recovering arapaima populations. In a set of four communities where the model was first tested, the arapaima population rebounded from ~ 50 arapaima/km^2^ before 1999 to ~ 500 ten years later (Fig. 3a). In the first few years, when the model was still being tested in those four communities, several fishers from neighboring communities requested that the Mamirauá Institute and other organizations implement the management model. This led to the involvement of growing numbers of communities and governmental and nongovernmental organizations, which implemented the model in a variety of social, ecological, and economic contexts. As of 2023, 407 fishing communities were authorized to harvest arapaima (Fig. 3b). Where studies have been done, arapaima populations are healthy and fisher revenues have increased (Arantes et al. 2006; Castello et al. 2009; Campos-Silva and Peres 2016; Petersen et al. 2016; Watson et al. 2021).

### Dilemmas in arapaima co-management

As the model expanded across communities, however, several endogenous and exogenous factors and processes emerged that threaten its effectiveness. As in many SSF, there is a legacy of lack of investments in basic infrastructure and ability to harvest, sell, assess fishery stocks, and implement and enforce management rules. At the community scale, one source of conflict involves the harvest quotas, with fishers from some fishing communities complaining that IBAMA sets quotas that are too low. This issue stems from absence of information to allow management to account for biophysical effects on the productivity of arapaima populations. The abundance of arapaima varies by orders of magnitude across lakes due to biophysical characteristics such as lake area, depth, type of vegetation, and hydrological connectivity (Arantes et al. 2013). This lack of understanding of biophysical effects forces IBAMA to set the quotas in the same way across all communities, even though a quota that may be sustainable in one community can be too small, or too large, in another.

Beyond ecological concerns, social dynamics also pose significant challenges for co-management. A key problem is rule breaking. Although IBAMA is legally mandated to patrol and enforce rules, officials are several hundred kilometers away from most fishing communities, unable to enforce rules. To address this challenge, the co-management model assigns patrolling and rule enforcement responsibilities to fishers (Peralta 2012; Franco et al. 2021). However, fishers lack formal authority to sanction rule offenders (Franco et al. 2021, 2025) and find it difficult to do so when rule offenders are their neighbors, friends, or relatives (Castello et al. 2009). An understanding of the drivers of rule compliance could help create the conditions to more successful co-management arrangements (Rodrigues et al. 2025). Yet, little is known about the topic, even though responsibility to manage fisheries and other natural resources is increasingly passed on from the government to rural communities (Brooks et al., 2012).

These challenges are compounded by social and ecological processes at the broader regional level. In recent decades, the Amazon River has experienced greater seasonal flooding, increased hydrological connectivity, and heightened interannual variability (Fleischmann et al. 2023). Continuous river meandering, especially along sediment-laden rivers (e.g., Amazon, Juruá), leads to major changes to the biophysical conditions of floodplain lakes (Nagel et al. 2022), ultimately affecting fish abundance in them (Granado-Lorencio et al. 2007; Nolan et al. 2009). The region has witnessed record-breaking floods and droughts in the last few years (Espinoza et al., 2022, 2024), making it impossible for many fishing communities to harvest their managed arapaima because of difficulties to access the lakes and transport the fish. How such hydrological and riverscape alterations are affecting the populations of arapaima and other species is unknown, but river hydrology is known to affect the dynamics of most floodplain fish populations (Cajado et al. 2023).

At the regional level, market dynamics also affect local-level rule enforcement, as patrolling costs are too high to be offset by selling prices. Selling prices are kept low by a few large businesses that control a market that is easily saturated by supply of unmanaged (i.e., illegally harvested) arapaima that is sold at low prices. This low profitability has become a key issue particularly since 2014 when Brazil entered an economic crisis that was exacerbated by the COVID-19 pandemic (Abessa et al. 2019; Levis et al. 2020; Pelicice and Castello 2021). These changes pushed fishing households further into poverty, thus fostering even more rule breaking (de Mattos et al. 2022). These various factors and processes highlight the need for a more integrated understanding of arapaima SSF.

## Integrating social–ecological feedbacks

### Conceptualizing arapaima small-scale fisheries

**Fig. 4:**
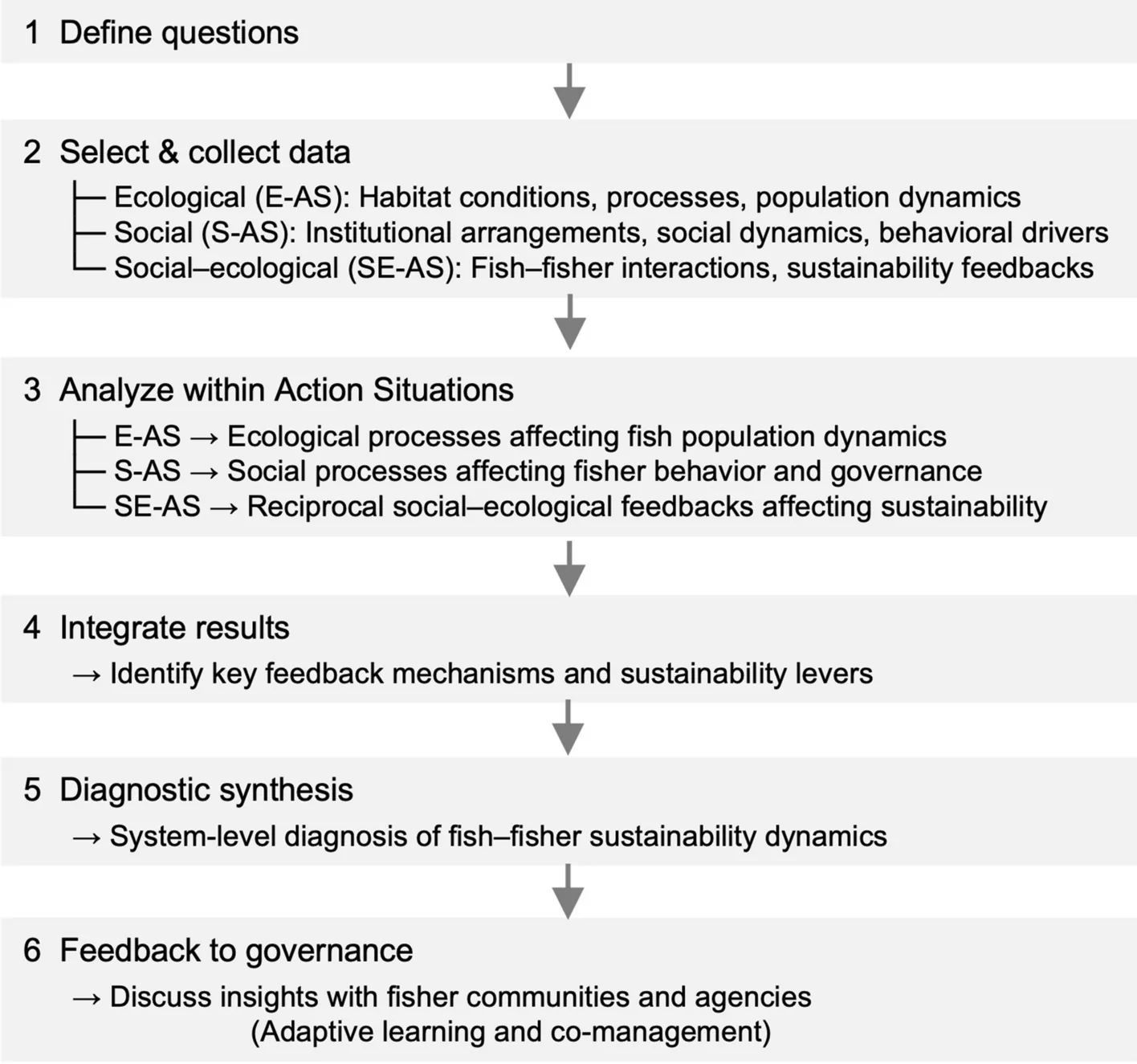
Operational workflow for diagnosing social–ecological feedbacks in small-scale fisheries. The workflow illustrates how the social–ecological action situation (SE-AS) framework (Fig. 1b) can be operationalized to assess reciprocal interactions between ecological dynamics and fisher behavior. The workflow proceeds from defining research questions to selecting ecological, social, and social–ecological data, followed by analysis within each Action Situation, integration of results, and diagnostic synthesis of key feedback mechanisms and sustainability levers. The final step emphasizes communicating findings to communities, managers, and policy actors to support adaptive learning and co-management. This workflow provides a generalizable template for implementing diagnostic assessment of sustainability in diverse small-scale fisheries contexts

Our approach to assess SSF for arapaima builds on the complementarity of fisheries and SES approaches. Our approach applies the SE-AS of Schlüter et al.’s framework to integrate ecological drivers of fish population dynamics with theories of fishers’ behavior and governance processes (Fig. 4). This integration accounts for the reciprocal fish–fisher feedbacks that drive sustainability (Fig. 1b). Similarly, we use the S-AS and E-AS of Schlüter et al.’s framework to adequately account for the dynamics of social and ecological systems, respectively (Fig. 1b), enabling balanced consideration of social and ecological dimensions. Figure 4 illustrates the conceptual structure and operational workflow of this integrative approach.

**Fig. 5:**
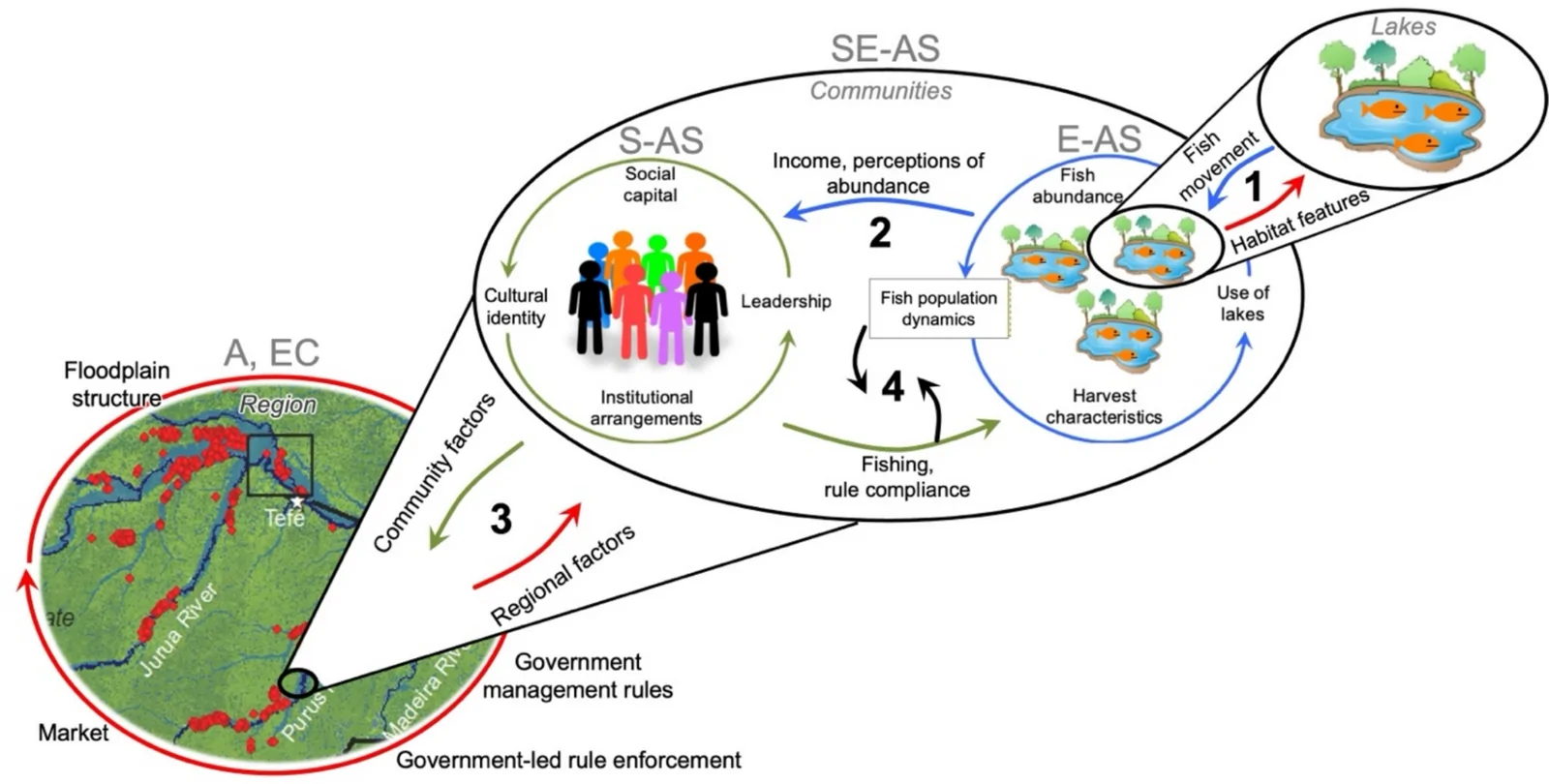
Schematic diagram of a multi-scalar approach to assess arapaima small-scale fisheries. The approach reflects the SE-AS framework proposed by Schlüter et al. (2019) to capture the reciprocal social and ecological feedbacks that are responsible for the emergence of system properties such as sustainability in fisheries. The sustainability of arapaima fisheries is hypothesized to emerge from interactions between two main sets of processes. These include the effects of lake habitat, movement, and hydrological connectivity on arapaima populations, which comprise the E-AS, and the effects of culture, community, and institutions on fisher behavior, which comprise the S-AS. The E-AS and S-AS are each influenced by larger-scale actors and processes in the ecosystem and regions where the arapaima fisheries take place. Numbers in bold represent the questions guiding research on these respective topics. This configuration of action situations (AS) can be hypothesized to be under the influence of other social and ecological factors, processes, and interactions involving actors (A) and ecosystem components (EC)

We framed the case of arapaima SSF as follows (Fig. 5). The arapaima and the fishers constitute the main SE-AS at the scale of the fishing community. They interact through reciprocal (two-way) processes involving the harvesting of arapaima and the perceptions and benefits derived from that harvesting. These feedbacks are influenced by sets of social and ecological factors and processes. Ecological processes influencing the dynamics of arapaima populations in floodplain lakes constitute the main E-AS. Social processes influencing the fishing behavior of fishers constitute the main S-AS. The dynamics of arapaima populations and fishers’ fishing behavior are also affected by regional-level processes. Because these factors and processes operate in complex ways and at multiple temporal and spatial scales, we used four guiding questions to encapsulate the key factors, processes, and interactions of interest (Fig. 5).

1. Does floodplain lake habitat affect the population dynamics of arapaima?

In the E-AS (Fig. 5), the ability of arapaima to sustain harvesting is expected to vary as a function of lake habitat and hydrological connectivity. As in other SSF marked by high diversity of species with small ranges (Mahon 1997), habitat selection theory indicates arapaima are heterogeneously distributed in the ecosystem due to their responses to local, smaller-scale ‘*habitat’* conditions in *‘lakes’* (Rose et al. 2001; Minns and Wichert 2005). The lakes are embedded in networks of river–floodplain channels and vegetated habitats that together determine hydrological *‘connectivity’* for fish and are influenced by interannual variability in *‘river hydrology’* (Fullerton et al. 2010; Castello et al. 2015a, b). Theory also indicates that, depending on connectivity, arapaima can move through such habitats, as they select, respond, and use habitat conditions that increase the fitness of their populations (Matthews 2012). Population ecology theory indicates that the ability of arapaima to sustain harvesting, which is correlated with *‘abundance of arapaima,’* depends on growth, reproduction, and natural and fishing mortality processes (Quinn and Deriso 1999). In lakes, rates of growth, reproduction, and natural mortality of arapaima are affected not only by local lake habitat structure, but also by connectivity of adjacent populations (e.g., metapopulations) and corresponding habitat structure at the regional level (Fig. 5). Lakes or fishing communities with different amounts of arapaima make available different amounts of arapaima for fishing communities to harvest, having thus the potential to influence *‘fisher behavior’* in different ways.

2. How do fishers’ perceptions of arapaima abundance in lakes and their livelihood dependence on fishing intersect with community leadership and culture to affect rule compliance?

In the S-AS (Fig. 5), fishers’ cooperative behavior in terms of compliance with the three management rules (i.e., size, season, and quota) is expected to depend on cultural aspects, community dynamics, and institutional arrangements. Collective action theory of common pool resources indicates that major advances in managing the arapaima come from implementation of property rights for fishing communities and management rules that constrain fisher behavior. However, fisher behavior is also shaped by observed ecological conditions as well as culturally embedded perceptions of fish abundance, reflecting an SE-AS process in which ecological dynamics directly inform social behavior, a linkage rarely accounted for (Ciumasu 2023). Most communities involved in arapaima co-management are located inside protected areas such as extractive or sustainable development reserves, where they have common property rights over fish resources (i.e., outsiders cannot fish; Campos-Silva and Peres 2016; Silva et al. 2025). Compliance with rules of size, season, and quota of harvest also can be expected to depend on *‘individual’*-, *‘community’*-, and *‘regional’*-level factors.

At the individual level, fishing is expected to affect fishers’ behavior by affecting their *‘livelihoods’* in terms of way of life satisfaction that includes *‘income’* they derive from fishing (Wilson et al. 2016; Matsumura et al. 2019). When the *‘perceptions’* of fishers indicate there is high abundance of arapaima, they may fish more; conversely, when they perceive the abundance of arapaima to be low, they may engage in collective action to modify community-level fishing behavior by adapting or creating new rules (Adams et al. 2003).

At the community level, the capacity of communities to develop collective action for management (i.e., *‘institutional capacity’*) to influence fishers’ behavior is expected to be affected by several factors in addition to property rights such as *‘leadership’* and ‘*culture’* (Tuler et al. 2008; Gutierrez et al. 2011). Culture is a poorly studied factor in fisheries that is expected to affect the behavior of fishers where fishing is part of a social identity (e.g., lobster fishing in the Gulf of Maine; Tengberg et al. 2012).

3. Is community-level rule compliance influenced by regional-level processes related to market characteristics, degree of interactions among communities, government-led rule enforcement, and perceptions of fairness and adequacy of governmental rules?

Regional-level factors of the S-AS (Fig. 5) can affect community-level institutional arrangements and rule compliance, including partnerships with technical institutions (advisory teams) with which the community organizations and fishers interact. ‘*Market*’ dynamics can influence income from fishing through price fluctuation variability in various price, types of commercialization arrangements, and logistical costs related to distance to markets. Persistent low market prices can limit the economic incentives for compliance, particularly when fishers perceive that management efforts and costs do not translate into improved livelihoods. In such conditions, where prices are consistently low and market opportunities limited, fishers may prioritize immediate income needs over long-term sustainability, weakening prospects for collective action (Young et al. 2006).

Community fishers can also be affected through interactions with neighboring communities about their *‘perceptions of government rules’* with respect to their enforcement, fairness, and adequacy (Falk et al. 2002). Perceptions of fairness are closely tied to collective choice arrangements, where fishers’ participation in rule-making fosters a sense of legitimacy and ownership (Ostrom 1990). When state enforcement of government rules is poor, which is typical in SSF, communities must shoulder the burden of monitoring and sanctioning. Because these activities are costly and socially risky, weak state enforcement can increase the likelihood of rule violations and weaken community-level cooperation in rule compliance (Ostrom 2005). Moreover, when arapaima fishers perceive government rules as unfair or inadequate—whether due to misalignment with local practices or lack of participation in decision-making—they may feel compelled to break rules more frequently. This reflects a common challenge in governance systems that fail to sufficiently engage local actors and consider local knowledge and social dynamics (Ostrom 2009), as in many SSF.

4. How do community rule compliance and habitat effects on arapaima populations interact to produce the emergent property of sustainable fish populations?

The process by which fishers harvest the arapaima constitute the reciprocal social–ecological feedback that determines the dynamics of arapaima SSF. This harvesting involves the catching of the arapaima (i.e., the social-to–ecological effect) as well as the benefits and perceptions of doing so (i.e., the ecological-to-social effect). The way these interactions take place determine whether arapaima fishing in any community is biologically sustainable. Fishers’ compliance with the three rules of size, season, and harvest quota directly influences arapaima population dynamics. This, in turn, determines whether biomass increases or decreases. The arapaima populations, in turn, affect fishers through the provision of food, income, and satisfaction, or by influencing their perceptions of arapaima abundance, which can prompt them to increase or decrease fishing effort. These fish–fisher feedbacks comprise the hotspot of social–ecological interactions that determine the productive capacity of arapaima populations to be sustained over time, whether they are driven by factors and processes that are endogenous or exogenous to the fisheries.

### Toward operationalizing the approach

We operationalized our approach using available guidance (e.g., Leslie et al. 2015; Partelow 2015) and adopting methods to collect and analyze data that permitted integration of diverse disciplines. We refer to *operationalization* here in the methodological sense, involving the steps of identifying specific data, indicators, and analytical techniques required for implementation. Operationalization in this context encompasses both quantitative and qualitative pathways. Quantitative indicators enable comparative and replicable analysis at broader scales, whereas qualitative methods (e.g., interviews, participant observation, and collaborative workshops) elucidate the cultural and institutional processes that shape fisher behavior. The two forms of data are integrated iteratively, ensuring that model development remains grounded in local meanings and lived experience.

To ensure a balanced emphasis on both social and ecological dimensions of arapaima SSF, we carefully selected and tailored our methods. A key priority was accounting for the reciprocal interactions between arapaima and fishers. The main methods and variables selected are summarized in Tables 1, 2, and 3. Because the operationalization of SES frameworks has remained largely elusive (e.g., Leslie et al. 2015; Partelow 2015; Vogt et al. 2015), we identified additional (though not an exhaustive list of) methods and variables that interested researchers can consider using in similar endeavors.

**Table 1:** Common methods to assess the ecological action situation (E-AS) arena to operationalize the approach to assess small-scale fisheries. Shown are examples of methods and instruments frequently used to characterize the environment and fish populations to understand habitat or ecosystem effects on the dynamics of the target fish population (Question 1). Methods used by our arapaima research are indicated (*). Most methods are found in key textbooks (Gallucci et al. 1996; Sparre and Venema 1999; Zale et al. 2013)

Component	Methods, instruments	Key variables
Habitat structure	Remote sensing*	Geographical extent and dynamics of aquatic habitats, land cover in the areas surrounding lakes
	Bathymetry*	Habitat depth, area, volume
Population size or fisheries status assessment	Fisheries landing data	Catch biomass per species for fishing trips, including measures of fishing effort and description of gear, place of fishing, rates of total, natural, and fishing mortality Age and size composition, sex ratios
	Fishers’ recalls of historical catches	Standardized catch per effort, species composition (Castello et al. 2023a, b; 2024)*
	Underwater acoustic survey	Fish biomass, abundance, distribution
	Population dynamics assessments (e.g., Virtual population analysis)	Estimates of past population sizes, per-capita birth and death rates
	Yield-per-recruit assessment	Yield responses to fishing mortality
	Fish counts*	Counts of arapaima (e.g., Castello 2004)
	Tagging, mark recapture*	Rates of immigration, emigration, population size
	N-mixture modeling*	Rates of survival, recruitment, immigration, emigration, population size
	Reading of growth increments in otoliths, scales; size–frequency analyses	Mean length at age, Von Bertalanffy growth parameters
	Temporal analyses of gonadal development, spawning events	Age and season of spawning
Movement	Tagging, mark recapture*	Patterns of movement, home range
	N-mixture modeling*	Rates of immigration and emigration
Age and growth	Interpretation or analysis of hard structures (e.g., scales, otoliths) of length–frequency data	Length at age, descriptors of somatic growth
Reproduction	Gonad inspection, analysis	Estimates of age, season, and body size at first spawning

**Table 2:** Common methods to assess the social action situation (S-AS) arena to operationalize the approach described here to assess small-scale fisheries. Shown are examples of community-level concepts and indicators for understanding the effects of institutional arrangements of rule compliance (Question 3) as well as determinants of rule compliance related to social capital, leadership, and cultural identity (Question 2). Methods used by our arapaima research are indicated (*). Indicators can be assessed using a variety of qualitative and quantitative methods such as surveys, interviews, and document analysis

Item	Description	Indicators
**Dimension related to social capital, leadership, and cultural identity (Question 2)**		
Social capital: trust	Degree of trust among fishers, leaders, and compliance monitors (Gilmour et al. 2011)*	Types of trust: evidence-based, feelings of social connectedness, trust in fair procedures, and personality predispositions (Stern and Baird 2015)
Livelihood engagement	Greater engagement indicates greater cohesiveness*	Degree of involvement in CBM activities, kinship ties, frequency of interactions with fishing group, leadership roles
Leadership	Characteristics of leaders; connections within and beyond communities (Sutton and Rudd 2014) *	Number of interactions with fishers; perceived effectiveness of leader; fishers’ level of trust in leader; adherence to management plan; fishers’ perceptions on direction, alignment and commitment (Drath et al. 2008)
Perceptions of fish population size	Fisher’s perception on the current size and trend of the fish population*	Fishers’ perceptions of current population size; fishers’ perceptions of population trends over time Likert scale on trend in arapaima population past 5 years (Decreased, Remained Unchanged, Increased)
Cultural identity	Role of shared experiences in reinforcing fishers’ identity as ‘a fisher’ (Greene 2004).*	Strength of fisher identity; collective self-esteem; family identity; institutional identity; sense of belonging (Mael and Tetrick 1992; Terry et al. 1999; Luhtanen and Crocker 1992)
Community arena	Fishers’ perceptions of CBM as ‘competitive’ or ‘cooperative’*	Fishers’ perceptions of CBM as competitive or cooperative (Xie et al. 2006)
Social norms	Fisher’s perception on informal expectations related to behaviors regarding rule adherence*	Perceived prevalence of rule violations; clarity of expected fishing behavior; expectations regarding rule compliance; tolerance for deviant fishing behavior; perceived autonomy in fishing behavior; perceived monitoring of fisher behavior (Based on Bicchieri 2016 and Gelfand et al. 2011)
**Dimension related to institutional arrangements (Question 3)**		
Social boundaries	If there are rules defining users’ boundaries; who can or not participate*	Existence and effectiveness of social boundary rules related to access, use, harvesting (Schlager and Ostrom 1992)
Physical boundaries	If there are rules defining boundaries for resource use and management*	Existence and effectiveness of boundaries, including types of limit demarcation, limit contestation, level of respect for limits (Cox et al. 2010b; Cole and Ostrom 2012)
Collective arrangements	Types of collective arrangements for resource use and management; recognition of group to define rules*	Existence and effectiveness of group(s) for use and governance, and degree of recognition of local organizations to govern resources (Pomeroy and Berkes 1997; McGinnis 2011)
Conflict resolution	Types of fishery-related conflicts; Means of conflict resolution; Level of gravity of conflicts*	Existence and effectiveness of conflict resolution mechanisms associated with rule compliance for size, season, and quota rules (Meinzen-Dick and Pradhan 2002; Dietz et al. 2003)
Control and monitoring	Types of control and monitoring; Authority to set and enforce rules*	Existence and effectiveness of control and monitoring systems for size, season, and quota rules (Cox et al. 2010b; McGinnis 2011)
Sanctions	Types of sanctions for noncompliance; graduation and efficacy of sanctions*	Existence and effectiveness of different types of sanctions for noncompliance with CBM rules (Cinner et al. 2012; Bicchieri 2016)
**Measuring rule compliance (Questions 2 and 3)**		
Rule compliance	Prevalence, frequency, and characterization of rule adherence*	Prevalence of rule violations; frequency of rule violations; types of rules most frequently violated; perceived levels of compliance (Chaves et al. 2021)

**Table 3:** Common methods to assess the social–ecological action situation (SE-AS) arena to operationalize the approach described here to assess small-scale fisheries. Shown are methods and instruments that can be used to assess interactions between fish and fishers to understand how rule compliance and habitat affect the productive capacity of arapaima populations (Question 4). Methods used by our arapaima research are indicated (*)

Component	Methods, instruments	Key variables
Fish–fishers’ feedbacks, including population dynamics and fishing effort	State-space modeling (Buckland et al. 2004; Schaub and Abadi 2011; Aeberhard et al. 2018; Kindsvater et al. 2018; Auger-Méthé et al. 2021; Newman et al. 2023)*	Intrinsic rate of population growth, density-dependent feedback
	Management strategy evaluation frameworks (Smith et al. 1999; Bunnefeld et al. 2011; Kaplan et al. 2021)	Depends on specific methods used to assess stock dynamics or fishery performance (see Table 1)

In essence, our approach to assess the sustainability of SSF implements methods to address our research questions (Table 1). In the E-AS, we assess environmental effects on fish population dynamics using four key methods. (1) Hydrology, remote sensing, and bathymetry characterize habitat structure and hydrological connectivity. (2) Biotelemetry understands arapaima movement and lake selection. (3) Population counts, harvest data, and N-mixture modeling provide insights into arapaima abundance and fishing mortality. And finally (4) hierarchical modeling estimates intrinsic population growth rates under varying harvesting and environmental conditions.

In the S-AS, the approach involves assessing community- and regional-level effects on management activities, including the fishing behavior of fishers. The approach involves four steps: (1) analyzing community arena and institutional arrangements (i.e., design principles) to understand the context of fisher rule compliance; (2) assessing the degree of rule breaking with the three harvesting rules (quota, season, and size limits); (3) examining relationships between the degree of rule breaking and resource dependence, perceptions of arapaima abundance, leadership, cultural identity, markets, degree of interaction among communities, level of government-led rule enforcement, and perceptions of fairness and adequacy of government rules; and (4) using regression models to model rule breaking as a function of candidate community- and regional-level factors and processes to identify the drivers of rule compliance. While Table 2 summarizes potential quantitative indicators, these are enriched by qualitative approaches (e.g., interviews, focus groups, and ethnographic observation) to capture context-specific meanings and social dynamics that cannot be reduced to numerical form.

In the SE-AS, our approach involves assessing the ability of arapaima populations to sustain harvesting under the effects of habitat and rule compliance. We built on the state-space models developed in the E-AS to incorporate key factors influencing sustainability. These include habitat characteristics and fisher behavior related to rule compliance (Brooks et al. In Press). The state-space models can be fitted to account for the multi-scalar nature of arapaima, whereby *lakes* are nested within (fishing) *communities*, which are in turn nested within *subregions* in the large floodplain system. The assessment can reveal the factors that exert strong influence on the productive ability of arapaima populations, and hence on the sustainability of arapaima SSF.

While assessments of reciprocal fish–fisher feedbacks are rare (Lade et al. 2015; Schlüter et al. 2019) and methods to model them are still in their infancy (e.g., Brooks et al. In Press), we anticipate similar and simpler methods can make them feasible in other SSF. Our assessment of fish–fisher feedbacks in arapaima SSF is enabled by the existence of unique population count data that are not available in most fisheries. Similar assessments in other data-poor SSF can be done using catch-per-unit-effort data (Kindsvater et al. 2018) or simpler conventional stock or fishery performance assessment methods (see Table 1; Gallucci et al. 1996; Sparre and Venema 1999). Such conventional assessment methods of stock or fishery performance can be linked to information on the drivers of rule compliance (from S-AS) via management strategy evaluation frameworks (Smith et al. 1999; Bunnefeld et al. 2011; Kaplan et al. 2021). Management strategy evaluation frameworks allow evaluation of management scenarios under a range of possible changes in fishing activities or behavior, which can be directly linked to the drivers of rule compliance.

Parallel to our methodology operationalization, we also engaged with fishers and management technical support units to jointly discuss and interpret the collected data and results it produced. Although the pandemic upended our ability to fully engage with resource managers and users, our collaboration aimed to make more robust science and foster actionable outcomes. To the extent possible, we have met, presented, and discussed our project with stakeholders almost every year from the start of the project. These meetings included community leaders, fishers, managers, and researchers. Discussions combined plenary sessions with small-group activities using the ‘World Café’ method (Löhr et al. 2020) to identify key issues and co-interpret preliminary results. Feedback from these sessions is being used to refine understanding of results and ensure local priorities are reflected in final results. By prioritizing the co-production of knowledge through participatory data collection methods, our approach fosters a collaborative environment where information, expertise, and perspectives are shared equally between researchers and community stakeholders. This collaboration creates regular feedback loops that build trust, encourages mutual learning, and integrates both local and scientific knowledge inform management practices. Figure 4 visually illustrates the research workflow of our approach to assess social–ecological interactions in SSF.

## Discussion

### Strengths of the approach to assess small-scale fisheries

Our approach to assess arapaima SSF addresses two key limitations of conventional fisheries and SES approaches. First, the approach provides balanced consideration of the multi-scalar social and ecological factors and processes that influence SSF. Second, our approach integrates fish population dynamics with models and theories of fishers’ behavior and community-level factors. This integration captures the reciprocal feedbacks between arapaima and their fishers, which are key to understanding the emergence of sustainability. Additionally, by considering the institutional arrangements that shape fishers’ behavior (e.g., clearly defined boundaries), our approach aligns with key principles of sustainable common pool resource management. Overall, our approach represents a step forward in the development of comprehensive assessment methods and offers insights into our current practice of integrating qualitative and quantitative data across multiple spatial and temporal scales.

Assessment approaches like ours that integrate key social–ecological feedbacks can contribute to improve the state of SSF. Given the scarcity of truly integrated approaches, a major source of uncertainty in fisheries management involves fisher behavior, which often leads to unplanned and unintended outcomes (Hilborn 2007; Fulton et al. 2011). Prior studies have shown that SSF governance could be improved through increased scientific understanding of the role played by human behavior, social norms, and human psychology (Yoeli et al. 2017; Cinner 2018; Partelow et al. 2021). In particular, there is a need to improve our understanding of how cultural and psychological drivers influence fisher behavior and are linked to other system variables and outcomes (Partelow 2018; Wagner 2019). Fisher perceptions of fish abundance is a key mechanism linking fish and fishers in reciprocal feedbacks. In arapaima, these feedbacks occur via harvesting and fishers’ counts of arapaima. In other fisheries, these feedbacks occur through harvesting and fishers’ perceptions of abundance based on that harvesting, as shown by Lade et al. (2015). Our approach fosters understanding of these critical connections by highlighting fish–fisher feedbacks and permitting identification of their linkages to other system components.

We anticipate that approaches like ours can help identify the levers of fishers’ behavior that can make fisheries sustainable. In a unique analysis accounting for reciprocal feedbacks between fish and fishers, Lade et al. (2015) showed that the collapse of the Baltic cod fishery arose from feedbacks involving psychological, economic, and regulatory drivers of fishers’ decision-making, which amplified fishing pressure and reinforced overfishing, in addition to ecological interactions. This interpretation reframed overfishing not as an independent cause of collapse, but as an outcome of coupled social–ecological feedbacks, contrasting with earlier explanations emphasizing only environmental deterioration and excessive harvest (Möllmann et al. 2009). Accounting for reciprocal fish–fisher feedbacks is necessary, because, as explained by systems theory, the property of self-regulation in systems requires feedback loops to emerge. In a generalized fishery, reciprocal feedbacks involve: (i) the effect of fishers on the exploited fishery stock via harvesting and (ii) the feedback effect of the fishery stock on the fishers via provisioning of income or fishers’ perceptions of stock abundance. In industrial fisheries, the conventional approach attempts to control the feedback mechanism regulating fishing behavior via enforcement of management rules based on scientific assessment of fishery stocks. In the absence of those assessments and rule enforcement in SSF, one must seek to understand fishers’ responses in terms of their own fishing behavior to changes in the fishery stock. Although further testing is needed, this notion that fish–fisher feedbacks is key to sustainability is consistent with social–ecological frameworks and the conventional fisheries approach, thus likely being broadly applicable.

Our approach identifies key social, institutional, and perceptual levers of fishers’ behavior that influence rule compliance and, ultimately, sustainability outcomes. These insights can directly inform policy and governance. Recognizing the importance of institutional arrangements, including effective monitoring and sanctioning, allows a more adaptive governance system, better grounded in reality (Ostrom 2005; Cox et al. 2010b). While many important questions can be posed about SSF governance, our experience describing and discussing the approach with fishers, community leaders, and technical personnel indicates they address critical issues. This has prompted interest from the Mamirauá Institute to use information on the drivers of illegal fishing in the joint determination of harvest quotas, with the aim of making them more biologically sustainable. For example, in the arapaima SSF, low fish prices due to market saturation and logistical barriers appear to constrains of rule compliance and management viability. Such insights may inform the design of policy interventions (e.g., decentralized marketing infrastructure; Ciumasu 2023). More importantly, the approach presented here can be used by governmental and nongovernmental organizations to guide regional efforts to address factors undermining the effectiveness of community-based management of SSF (Cavole et al. 2015; Quimbayo et al. 2022). For example, if trust in local leadership is a strong predictor of rule compliance, then investments in leadership capacity can serve as an important governance lever within community-based management systems, where formal top-down enforcement is limited or absent.

### Limitations of the approach

Our approach faces two main limitations. The first is requiring substantial research efforts to be implemented in full, particularly in terms of collecting data. That level of research effort, including detailed time series of catch and counts, is rare in SSF and unfeasible for most research and management organizations in the regions where SSF abound. Because the approach combines quantitative and qualitative data, effective implementation demands interdisciplinary teams and sufficient time to integrate insights across methods. Nevertheless, even partial applications can draw on qualitative evidence to interpret or validate limited quantitative data, maintaining analytical balance while adapting to resource constraints. For that reason, our approach can guide future research efforts that can alternatively apply it in scaled down form. As we have shown, improved assessment of SSF will come from consideration of the key fish–fisher interactions and social and ecological dimensions that influence sustainability. Therefore, interested researchers may wish to prioritize not large volumes of data on all aspects of SSF, but rather data on the key factors and processes.

As formulated, our approach primarily addresses the ecological dimension of sustainability, namely the biological productivity of exploited fish populations. Other ecological aspects, such as hydrological variability, floodplain vegetation dynamics, or predator–prey interactions, could also be incorporated into future applications of the framework to enhance diagnostic breadth. However, sustainability also depends on social and economic dimensions, such as institutional capacity, rule compliance, and the financial viability of management. For example, several fishers involved in arapaima management report economic viability as a critical factor influencing their decisions to continue or not to do management, particularly considering the high costs of doing lake patrolling and rule enforcement. Future research should build on this foundation by developing methods to assess the social and economic conditions that enable or constrain long-term sustainability in SSF.

### Conclusions

Our approach is one of many steps toward achieving a truly integrated assessment of SSF. By bridging ecological and social perspectives, our approach advances our understanding of sustainability dynamics. The approach we described builds on Schlüter et al.’s framework to merge key strengths of conventional fisheries and SES approaches, allowing to directly link the dynamics of exploited fish populations to the self-organization of fishers and related exogenous factors and processes. While there are ways to improve upon this approach, our hope is that our description and analysis of it will foster the development of more integrated assessment approaches for SSF.

Diagnostic approaches like ours can be useful tools to foster sustainability in SSF. Because SSF are numerous, they offer unparalleled research possibilities through space-for-time substitution approaches. As increasingly done in recent years (e.g., Cinner et al. 2012; Leslie et al. 2015; Arantes et al. 2022), implementation of systematic research efforts has identified important principles of SSF governance. By centering on the identification of sustainability levers—those variables whose influence cascades through the system—our approach provides a blueprint for diagnosing where and how governance efforts can be most effective. Future work can apply this diagnostic framework to other SSF systems to uncover context-specific levers and support evidence-based policy design.
